# Influence of Genetic Variation in *COMT* on Cisplatin-Induced Nephrotoxicity in Cancer Patients

**DOI:** 10.3390/genes11040358

**Published:** 2020-03-27

**Authors:** Bram C. Agema, Stijn L.W. Koolen, Mirjam de With, Nadia van Doorn, Niels Heersche, Esther Oomen-de Hoop, Sabine Visser, Joachim G.J.V. Aerts, Sander Bins, Ron H.N. van Schaik, Ron H.J. Mathijssen

**Affiliations:** 1 Department of Clinical Chemistry, Erasmus University Medical Center, 3015 GD Rotterdam, The Netherlands; m.dewith@erasmusmc.nl (M.d.W.);; 2 Department of Medical Oncology, Erasmus MC Cancer Institute, Erasmus University Medical Center, 3015 GD Rotterdam, The Netherlandsn.vandoorn@erasmusmc.nl (N.v.D.); n.heersche@erasmusmc.nl (N.H.); e.oomen-dehoop@erasmusmc.nl (E.O.-d.H.); s.bins@erasmusmc.nl (S.B.);; 3 Department of Clinical Pharmacy, Erasmus University Medical Center, 3015 GD Rotterdam, The Netherlands; 4 Department of Pulmonology, Erasmus University Medical Center, 3015 GD Rotterdam, The Netherlands; s.visser.2@erasmusmc.nl (S.V.);; 5 Department of Pulmonology, Amphia Hospital, 4818 CK Breda, The Netherlands

**Keywords:** cisplatin, acute kidney injury, *COMT*, nephrotoxicity, SNP

## Abstract

Cisplatin is a chemotherapeutic agent widely used for multiple indications. Unfortunately, in a substantial set of patients treated with cisplatin, dose-limiting acute kidney injury (AKI) occurs. Here, we assessed the association of 3 catechol-O-methyltransferase (*COMT)* single nucleotide polymorphisms (SNPs) with increased cisplatin-induced nephrotoxicity. In total, 551 patients were genotyped for the 1947 G>A (Val158Met, rs4680), c.615 + 310 C>T (rs4646316), and c.616–367 C>T (rs9332377) polymorphisms. Associations between these variants and AKI grade ≥3 were studied. The presence of a homozygous variant of c.616-367C>T was associated with a decreased occurrence of AKI grade 3 toxicity (*p* = 0.014, odds ratio (OR) 0.201, 95% confidence interval (CI) (0.047–0.861)). However, we could not exclude the role of dehydration as a potential cause of AKI in 25 of the 27 patients with AKI grade 3, which potentially affected the results substantially. As a result of the low incidence of AKI grade 3 in this dataset, the lack of patients with a *COMT* variant, and the high number of patients with dehydration, the association between *COMT* variants and AKI does not seem clinically relevant.

## 1. Introduction

Cisplatin is a widely used cytostatic agent that interferes with DNA replication by binding within (and between) DNA strands resulting in the inhibition of protein synthesis and DNA replication. It is approved for the treatment of many types of cancer such as testicular seminoma, non-small cell lung cancer, and head and neck cancer [1]. Common toxicities associated with cisplatin treatment include nephrotoxicity, ototoxicity, and leukopenia, of which nephrotoxicity is the main cause of dose reduction or even termination of systemic therapy. Treatment-related acute kidney injury (AKI) occurs in 20–30% of cisplatin-treated patients and is dose-dependent [2]. 

In a recently published case report, the potential role of catechol-O-methyltransferase (COMT) on cisplatin-induced nephrotoxicity was suggested [3]. Single nucleotide polymorphisms (SNPs) in five candidate genes, of which two are SNPs in *COMT,* were genotyped in a young woman who developed severe irreversible cisplatin-induced nephropathy. Both *COMT* polymorphisms were already known to be associated with cisplatin induced ototoxicity [4,5] and were homozygous polymorphic in this patient (c.615 + 310C>T (rs4646316) and c.616-367C>T (rs9332377)) [3]. As COMT is an enzyme that inactivates catecholamines by transferring a methyl group of S-adenosyl methionine (SAM) into a catecholamine neurotransmitter (such as dopamine, epinephrine, and norepinephrine), the authors hypothesized that the mechanism of cisplatin-induced nephrotoxicity could be mediated by increased SAM concentrations. This theory is supported by data from a mouse model study where cisplatin toxicity was 3- to 6-fold increased when SAM and cisplatin were administered concomitantly, compared to cisplatin administration alone [6]. The association between these two *COMT* polymorphisms and cisplatin-induced nephrotoxicity has not been validated in further clinical research. 

Another polymorphism in the *COMT* gene, the *COMT* 1947G>A (rs4680), is also known to affect COMT function [7]. This SNP is associated with response to other drugs [8,9,10,11], but has not been tested yet for the association with cisplatin-induced AKI.

Here, we studied the association between these three *COMT* polymorphisms and cisplatin-induced nephrotoxicity in a large cohort of cancer patients in order to assess the clinical relevance of these SNPs and their potential use as predictor in this context.

## 2. Methods

### 2.1. Study Design

Patients treated with cisplatin at the Erasmus MC Cancer Institute according to standard of care between January 2004 and April 2019 and who provided informed consent to prospectively perform DNA genotyping in their blood samples (Erasmus MC institutional review board study number MEC 02.1002) were included in this genetic association study. Patients treated with cisplatin and pemetrexed in the PERSONAL (PEmetrexed and biomaRkerS: an observatiONAL) study were also included in this analysis [12]. All patients were advised to drink two liters of water a day during cisplatin treatment and four liters of glucose/NaCl/KCl/MgSO_4_ solution was administered intravenously in 24 h on the day of cisplatin administration. The most recent plasma creatinine concentrations within two weeks prior to the first cisplatin administration were used as a baseline value. The highest plasma creatinine concentration within the period of first cisplatin administration until three weeks after the second 3-weekly administration was used for statistical analysis. Patients were excluded if baseline or on-treatment creatinine concentrations were not available or if the cisplatin dosage was not clearly recorded. 

### 2.2. Classification of Endpoints and Adverse Events

AKI was graded according to the Common Terminology Criteria of Adverse Events (CTCAE) version 4.03 (Table 1) [13]. AKI grade 3 or higher was interpreted as ‘severe AKI’, and was, therefore, the primary outcome in this study.

Of patients with AKI grade 3 or higher, the patient files were studied for potential confounders, such as dehydration and renal and post-renal pathology and recovery. When the plasma creatinine concentrations of these patients did not return to baseline +20 μmol/L in 2 years after cisplatin administration, this was classified as irreversible cisplatin-induced nephrotoxicity.

### 2.3. DNA Isolation and TaqMan® Genotyping

DNA was isolated using the MagNA Pure 96 Instrument (Roche Diagnostics GmbH, Rotkreuz, Switzerland), from 500 µL of the whole-blood specimens using the MagNA Pure 96 DNA and Viral Large Volume Kit (Roche Diagnostics GmbH, Rotkreuz, Switzerland). The selected *COMT* SNPs were genotyped using predesigned Drug Metabolism Enzymes TaqMan® allelic discrimination assays on the Life Technologies TaqMan®7500 system (Applied Biosystems, Life Technologies Europe BV, Bleijswijk, The Netherlands). Assay IDs are listed in Table 2. Each assay contains assay-specific primers and allele-specific minor groove binding probes labelled with VIC^™^ and FAM™ fluorescent dyes. Using these assays, TaqMan®GTXpress Master Mix and 20 μg genomic DNA, the polymerase chain reactions were performed in a reaction volume of 12 μL. The thermal profile of the TaqMan® qPCR reaction consisted of 40 cycles of denaturation (95 °C for 20 s), annealing (92 °C for 3 s), and extension (60 °C for 30 s). Genotypes were determined by measuring allele-specific fluorescence using the TaqMan®7500 software v2.3 for allelic discrimination.

### 2.4. Statistical Analysis

Distribution of genotypes was tested for Hardy Weinberg equilibrium; if p < 0.05, the genotype was excluded from analysis. The minor allele frequency (MAF) was also determined and compared to the MAF in the literature (Table 2).

Associations of the SNPs with the endpoints were tested in the dominant model; heterozygous variant (HT) and homozygous variant (HM) versus wildtype (WT), or the recessive model; and HM versus HT and WT. Prior research was reviewed to determine if the variant affected the SNP in a recessive or dominant manner. *COMT* 616-367C>T and 1947 G>A were tested in the dominant model [4,15]. *COMT* 615+310 C>T was tested using the recessive model [4]. For both the dominant and recessive models, the chi-squared test or Fisher’s exact test was used, depending on the cell counts of the contingency tables. When the cell count in any of the tables was less than 10, Fisher’s exact test was used.

Univariable associations of AKI with *COMT* polymorphisms *p* < 0.1 were considered for multivariable logistic regression analysis together with baseline variables such as age, gender, and cisplatin dosage, if possible. When Fishers’ exact test was used, no multivariable analysis was performed due to the large influence of chance on the outcome.

## 3. Results 

### 3.1. Patients

A total of 551 patients, with different malignancies, were included in this study. All genotypes were in Hardy–Weinberg equilibrium (Table 2). Baseline characteristics of these patients are depicted in Table 3. None of the included patients had renal dysfunction at baseline. Compared with baseline, median increase in plasma creatinine values was 10 μmol/L (interquartile range (IQR) 3–21 μmol/L) during treatment. AKI grade 3 occurred in 27 patients (5%).

### 3.2. Effect of COMT SNPs on Nephrotoxicity

All tested associations between the SNPs and AKI grade 3 are displayed in Table 4. *COMT* c.616-367C>T was significantly associated with less AKI grade 3 when performing a dominant analysis (CT + TT vs. CC, *p* = 0.014, odds ratio (OR) 0.201 95% confidence interval (CI) (0.047–0.861)). Age ≥65 years was univariably associated with the incidence of AKI grade 3 (*p* = 0.025, OR 2.464, 95% CI (1.095–5.542)) but dosage ≥80 mg/m^2^ and gender were not (Table 4). Association between the SNPs and Δestimated glomerular filtration rate (eGFR) was also tested (Supplementary Table S1).

Upon analysis of patient files of the patients with AKI grade 3, the creatinine concentration did not return to baseline in four out of 27 patients. In 25 out of 27 patients that had AKI grade 3, dehydration could not be excluded as a potential cause of AKI (Table 3). 

*COMT* 1947G>A and *COMT* c.615 + 310C>T were not significantly associated with AKI. 

None of the associations could be tested multivariably as all genetic associations were tested using Fishers’ exact test.

## 4. Discussion

This was the first study in which the association of *COMT* SNPs with cisplatin-induced AKI was assessed. Although we found a significant association between *COMT* c.616-367 C>T and cisplatin-induced AKI grade 3, we cannot rule out that this result was affected by dehydration, which we could not exclude in 25 of 27 patients. Insufficient data regarding hydration state was available as the data collection was performed retrospectively. The association of dehydration could, therefore, not be statistically tested in this cohort. However, as 25 patients with AKI grade 3 were potentially dehydrated according to their patient files, this could indicate that dehydration is a more important factor in the development of AKI than *COMT* SNPs. 

Despite this large cohort of patients treated with cisplatin, the occurrence of AKI grade 3 was insufficient to perform the chi-square test, and, therefore, did not comply with the requirements for multivariable analysis as stated in the methods section. An analysis using any AKI was not performed as this would not be clinically relevant. The occurrence of AKI grade 1 or 2 would not lead to dose reduction or treatment delay. As all but four patients with AKI grade 3 ultimately had complete recovered renal function, the underlying pathophysiological mechanism seems to point at a prerenal cause of AKI, which was most likely caused by dehydration. Moreover, based on the physiological mechanism, one would expect that patients harboring this polymorphism would be at more risk of developing AKI grade 3, rather than at reduced risk as we found. Additionally, the other investigated SNPs in *COMT* were not significantly associated with AKI grade 3. These data do, therefore, not provide a rationale for pre-emptive genotyping of *COMT*.

Of the patients who developed AKI grade 3, 52% were diagnosed with head or neck tumors (Table 3). Thirteen out of these 14 patients with this tumor type were treated by chemoradiotherapy (including cisplatin) which caused mucositis in up to 97% of the patients [16]. Oral mucositis can cause severe pain, which complicates drinking and is, therefore, a causal factor for dehydration. The fact that head and neck tumors are overrepresented in this group underlines the plausibility that AKI grade 3 in most of the patients was caused by dehydration. The standard use of prehydration and posthydration schemes show that many efforts are being made to prevent cisplatin-induced nephrotoxicity. Nonetheless, these efforts do not guarantee that the kidney function will remain stable during treatment.

The size of the studied patient population, which was prospectively identified, allowed us to study a relatively large number of patients with cisplatin-induced AKI. However, the retrospective collection of clinical data comes with several limitations. As we studied plasma creatinine concentrations, exact pathophysiological mechanisms of kidney injury could not be retrieved. In most patients, dehydration had to be determined by anamnestic information and (the time of) recovery after rehydration. Data on possible confounders such as fluid intake, co-medication, diarrhea, and nausea were not available and, due to the small sample size, could not be adjusted for in a multivariate analysis. However, these factors as a possible cause of AKI must be taken into account during cisplatin treatment. Other causes of AKI grade 3 have not been investigated regularly and urinalysis was performed only in a minority of patients. Moreover, there was large heterogeneity in the interval between blood draws, since these were only regularly scheduled in advance of each chemotherapy cycle and additional blood draws were executed based on clinical judgement of symptoms and hydration status. Therefore, temporary and asymptomatic elevations in plasma creatinine might have been missed in this study. 

Cisplatin-induced nephrotoxicity is currently mostly prevented with hyperhydration, magnesium supplementation or mannitol-induced forced diuresis, whereas none of these measures will guarantee that kidney function will remain stable [17]. Amifostine and cimetidine exert serious side effects and offer only a partial degree of nephroprotection against cisplatin nephrotoxicity [18,19,20,21]. Erlotinib has been suggested to have a beneficial effect on cisplatin nephrotoxicity by competitively inhibiting the OCT2 enzyme. This has not been validated yet [22]. So far, no perfect prevention is present, which renders the search for predictors of important dose limited toxicities of cisplatin still relevant. 

In conclusion, we found that *COMT* c.616-367 C>T is significantly protectively associated with the incidence of AKI grade 3 during cisplatin treatment. Despite the considerable size of this cohort, there was insufficient power and lack of data on hydration state to investigate the extent in which possible confounders such as dehydration affected this result. This study did not provide a rationale for pre-emptive genotyping of *COMT* SNPs to predict AKI in patients treated with cisplatin. The retrospective nature of this study necessitates prospective validation.

## Figures and Tables

**Table 1:** Definition of Common Terminology Criteria of Adverse Events (CTCAE) and Kidney Disease: Improving Global Outcomes (KDIGO) grading of acute kidney disease (AKI) in adults [13,14].

CTCAE		KDIGO		
Grade	Serum creatinine	Stage	Serum creatinine	Urine output
1	1.5–2.0 × baseline; OR ≥0.3 mg/dL (26.5 µmol/L) increase	1	1.5–1.9 × baseline; OR ≥0.3 mg/dL (26.5 µmol/L	<0.5 mL/kg/h for 6–12 h
2	2.0–3.0 × baseline	2	2.0–2.9 × baseline	<0.5 mL/kg/h for ≥12 h
3	>3.0 × baseline; OR > 4.0 mg/dL (353.6 µmol/L) increase; OR Hospitalization indicated	3	3.0 x baseline; OR ≥ 4.0 mg/dL (353.6 µmol/L) increase; OR Initiation of renal replacement therapy	<0.3 mL/kg/h for ≥24 h; OR anuria for ≥12 h
4	Life-threatening consequences; OR Dialysis indicated			
5	Death			

**Table 2:** Details and distributions of the studied single nucleotide polymorphisms (SNPs).

Gene	rs number	Variant	Assay ID	WT	HT	HM	MAF	HW
*COMT*	rs4646316	**c.615 + 310C>T**	C__29193982_10	316	204	31	24%	0.80
	rs9332377	**c.616-367C>T**	C__29614343_10	400	140	11	15%	0.76
	rs4680	**c.472G>A (Val158Met)**	C__25746809_50	117	282	152	53%	0.51

WT, wildtype; HT, heterozygous variant; HM, homozygous variant; MAF, minor allele frequency; HW, Hardy-Weinberg P value

**Table 3:** Patient characteristics.

Characteristics	Controls, *n* = 524	AKI grade 3 (*n* = 27)
Gender (n)		
- Male	310 (59%)	20 (74%)
- Female	214 (39%)	7 (26%)
Age (years)		
- Median (IQR)	57 (45–65)	62 (57–66)
Primary tumour (n)		
- Pulmonary	139 (27%)	4 (15%)
- Gastrointestinal	121 (23%)	2 (7%)
- Testicular	71 (14%)	1 (4%)
- Gynaecological	66 (13%)	3 (11%)
- Head / neck	56 (11%)	14 (52%)
- Other	71 (14%)	3 (11%)
Baseline plasma creatinine concentrations		
- Median (IQR) (μmol/L)	69 (59–81)	70 (64-77)
- Median (IQR) (mg/dL)	0.78 (0.67–0.92)	0.79 (0.72–0.87)
eGFR		
- Median CKD-EPI (IQR) (mL/min)	98 (85–106)	95 (81–99)
Peak creatinine concentrations		
- Median (IQR) (μmol/L)	81 (69–96)	131 (117–166)
- Median (IQR) (mg/dL)	0.92 (0.78–1.09)	1.48 (1.32–1.88)
eGFR		
- Median CKD-EPI (IQR) (mL/min)	87 (70–99)	44 (35–51)
Dehydration as potential cause of AKI (n)	N/A	25 (93%)
Irreversible creatinine augmentation (n)	N/A	4 (15%)
Cisplatin dosage (n)		
- <50 mg/m^2^	119 (23%)	10 (37%)
- 50–79 mg/m^2^	298 (57%)	12 (44%)
- ≥ 80 mg/m^2^	107 (20%)	5 (19%)

**Table 4:** Associations of AKI grade 3 with SNPs and confounders and the number of AKI grade 3 per patient group.

Endpoint	Factor	Groups (AKI grade 3/patients)	Univariable	
			OR ^ɣ^ (95% CI)	*p*-value
**AKI grade 3**	***COMT* (615+310C>T)**	TT (4/31) vs CC + CT (23/520)	3.201 (1.034-9.912)	0.058 *
	***COMT* (616-367C>T)**	CT + TT (2/151) vs CC (25/400)	0.201 (0.047-0.861)	0.014 *
	***COMT* (1947G>A)**	GA + AA (21/434) vs GG (6/117)	1.063 (0.419-2.697)	0.814 *
	**Age ≥ 65**	≥65 (10/111) vs <65 (17/440)	2.464 (1.095-5.542)	0.025
	**Dosage ≥ 80mg/m^2^**	≥80 (7/112) vs <80 (20/439)	1.397 (0.575-3.391)	0.464 *
	**Gender**	Male (20/310) vs Female (7/221)	1.972 (0.820-4.746)	0.159 *

* Fisher’s exact test, ^ɣ^ Odds ratio of ‘Groups’.

## Supplementary Materials

The following are available online at https://www.mdpi.com/2073-4425/11/4/358/s1, Table S1: Associations of ΔeGFR with SNPs and confounders.
