# Supplementary material for: Influence of Genetic Variation in COMT on Cisplatin-Induced Nephrotoxicity in Cancer Patients
Source: Genes (Basel). 2020 Mar 27;11(4):358. doi: 10.3390/genes11040358 (PMC7230333; doi:10.3390/genes11040358)
Supplement: Supplementary file 1 [file genes-11-00358-s001.pdf]

genes MDPI

Supplementary Table S1: Associations of ΔeGFR with SNPs and confounders.

Endpoint	Factor	Genotype (mean ΔGFR from baseline)	Univariable	
			**ΔeGFR (95% CI)**	***p*-value**
ΔeGFR (CKD-EPI)	*COMT* (615+310C>T)	TT (−13.19) vs CC + CT (−13.71)	0.518 (−5.373–6.409)	0.863
	COMT (616-367C>T)	CT + TT (−12.11) vs CC (−13.64)	−1.529 (−4.570–1.511)	0.324
	COMT (1947G>A)	GA + AA (−13.18) vs GG (−13.35) ≥65	−0.172 (−3.491–3.147)	0.919
	Age > 65	(−16.47) vs <65 (−12.40)	4.072 (0.705–7.439)	0.018
	Dosage ≥ 80mg/m^2	≥80 (−11.77) vs <80 (−13.59)	−1.821 (−5.191–1.549)	0.289
	Gender[+]	Male (−12.44) vs Female (−14.38)	−1.944 (−4.708–0.821)	0.168
